# Draft Genome Sequence of the Thermophilic Bacterium Bacillus licheniformis SMIA-2, an Antimicrobial- and Thermostable Enzyme-Producing Isolate from Brazilian Soil

**DOI:** 10.1128/MRA.00106-20

**Published:** 2020-04-23

**Authors:** Samara Pinto Custodio Bernardo, Albert Remus R. Rosana, Adriane Nunes de Souza, Sorina Chiorean, Meire Lelis Leal Martins, John C. Vederas

**Affiliations:** a Food Technology Laboratory, State University of North Fluminense, Campos dos Goytacazes, Rio de Janeiro, Brazil; b Department of Chemistry, University of Alberta, Edmonton, Alberta, Canada; University of Rochester School of Medicine and Dentistry

## Abstract

Bacillus licheniformis SMIA-2, a thermophilic and thermostable enzyme-producing bacterium, is found to be active against several strains of Staphylococcus aureus and several *Bacillus* species. Here, we report the 4.30-Mbp draft genome and bioinformatic predictions supporting gene inventories for amylase, protease, cellulase, xylanase, and antimicrobial compound biosynthesis.

## ANNOUNCEMENT

*Bacillus* sp. SMIA-2 is an important Brazilian strain for the production of industrially relevant thermostable enzymes such as amylases (1), xylanases (2), proteases (3), and cellulases (4, 5) utilizing diverse industrial fermentation substrates such as whey, sugarcane bagasse, corn steep liquor, and food waste (6, 7). SMIA-2 was isolated in 2001 from the soil in Rio de Janeiro, Brazil. Serially diluted soil was plated on tryptone-saline-yeast extract agar (TSYA) and incubated at 65°C for 24 h, and the single-colony isolate SMIA-2 was maintained on TSYA (8). The strain was phylogenetically categorized in thermophilic *Bacillus* group 5, with 94% similarity to *Bacillus caldoxylolyticus* (GenBank accession number AH010483.2) (8). Our resequencing of the 16S rRNA gene (MN645931) revealed that SMIA-2 is 100% identical to the type strain Bacillus licheniformis Gibson 46. We embarked on sequencing the genome of SMIA-2 because it is an important strain used in agricultural waste fermentation (6), laundry detergent development (9), and thermostable enzyme production (4–7) for second-generation bioethanol production in Brazil.

Genomic DNA was purified from a 12-h culture grown at 50°C in brain heart infusion broth (at 200 rpm) by using the DNeasy blood and tissue kit (Qiagen) following the manufacturer’s protocol for Gram-positive bacterial DNA extraction. DNA was quantified using a Qubit 2.0 fluorometer, and sequencing libraries were created using the Nextera XT DNA library preparation kit (Illumina, San Diego, CA) and sequenced using the NextSeq reagent kit (2 × 150 bp). Default parameters were used for all software unless otherwise specified. FastQC v0.11.8 (http://www.bioinformatics.babraham.ac.uk/projects/fastqc) was used to inspect the quality of the sequences, and quality trimming was based on Phred quality scores of 20 with SolexaQA v3.0 (10). Trimmed reads were *de novo* assembled using IDBA-UD v1.1.1 (11), implemented in the Microbial Genomes Atlas (MiGA) Pipeline v0.3.6.2 (12). The draft genome sequence was annotated using the NCBI PGAP v4.8 (13). Taxonomic classification was established using MiGA v0.5.0.0 (12), the average nucleotide identity (ANI) was calculated using the OrthoANIu v0.90 server (14), and digital DNA-DNA hybridization (dDDH) values were determined using the Genome-to-Genome Distance Calculator (GGDC) v2.1 server (15).

The SMIA-2 genome showed an ANI of 99.71% and alignment fraction of 0.97 with *Bacillus* sp. strain H15-1, whereas a comparison with the closest type strain, B. licheniformis Gibson 46, yielded an ANI of 99.57% (alignment fraction, 0.95), supporting the placement of SMIA-2 in the species B. licheniformis. SMIA-2 is a novel strain, as revealed by dDDH values of <79% (formula 2). Paired-end sequencing yielded 46,616,926 reads (233× coverage). The draft genome is 4,292,816 bp in 34 contigs (*N*_50_, 317,403 bp), with a G+C content of 45.85%.

Genome annotation detected 4,322 coding sequences, 11 rRNA genes, and 79 tRNAs. The genome contains gene inventories supporting thermostable enzyme production, while a total of 13 gene clusters for putative biosynthetic secondary metabolites were predicted using antiSMASH v5 (16). A summary of the genome scan highlights 5 of the 10 clusters (Table 1). Lastly, the thermostable enzymatic activities of SMIA-2 (1–4) can be supported by gene inventories, including 5 amylase genes, 13 loci for xylose metabolism, 55 protein degradation-associated loci, and 3 cellulolytic enzyme loci under a putative cellulosome complex (17).

**TABLE 1:** Summary of antiSMASH results for Bacillus licheniformis SMIA-2

Predicted biosynthetic metabolite	Contig no.	Position within contig (nucleotide range)	% similarity (known cluster)
NRPS^*a*^	4	189027–243708	100 (lichenysin biosynthetic gene cluster)
NRPS	5	147101–175615	53 (fengycin biosynthetic gene cluster)
Lassopeptide	9	111876–134337	0 (no known biosynthetic gene cluster)
Lanthipeptide	9	198527–225488	100 (lichenicidin biosynthetic gene cluster)
NRPS	21	1–20181	46 (bacillibactin biosynthetic gene cluster)

a NRPS, nonribosomal peptide synthetase.

### Data availability.

The whole-genome project for Bacillus licheniformis SMIA-2 has been deposited in DDBJ/ENA/GenBank under accession number JAACZZ000000000. The version described in this paper is the first version (JAACZZ010000000), under BioProject number PRJNA602865, BioSample number SAMN13909444, and Sequence Read Archive (SRA) number SRX7638223.
